# The Diagnostic Performance of Multiparametric Ultrasound in the Qualitative Assessment of Inconclusive Cervical Lymph Nodes

**DOI:** 10.3390/cancers15205035

**Published:** 2023-10-18

**Authors:** Katharina Margherita Wakonig, Steffen Dommerich, Thomas Fischer, Philipp Arens, Bernd Hamm, Heidi Olze, Markus Herbert Lerchbaumer

**Affiliations:** 1 Department of Otorhinolaryngology, Charité-Universitätsmedizin Berlin, Corporate Member of Freie Universität Berlin, Humboldt-Universität zu Berlin, and Berlin Institute of Health, Campus Virchow Klinikum and Campus Charité Mitte, Charitéplatz 1, 10117 Berlin, Germany; steffen.dommer@charite.de (S.D.); philipp.arens@charite.de (P.A.); heidi.olze@charite.de (H.O.); 2 Department of Radiology, Charité-Universitätsmedizin Berlin, Corporate Member of Freie Universität Berlin, Humboldt-Universität zu Berlin, and Berlin Institute of Health, Charitéplatz 1, 10117 Berlin, Germany; thom.fischer@charite.de (T.F.); bernd.hamm@charite.de (B.H.); markus.lerchbaumer@charite.de (M.H.L.)

**Keywords:** mpUS, multiparametric ultrasound, CEUS, SWE, head and neck

## Abstract

**Simple Summary:**

Enlarged cervical lymph nodes (CLNs) are routinely evaluated for distinction between benign infectious enlargements and malignant diseases. Ultrasound (US) is the first-line imaging modality for the assessment of CLNs. The aim was to investigate the diagnostic performance of the addition of the multiparametric applications shear wave elastography (SWE) and contrast-enhanced US (CEUS) to B-mode US and color-coded duplex sonography (CCDS). Our results show that the diagnostic performance of multiparametric US (mpUS) is significantly higher than that of B-mode US and CCDS. MpUS may help to distinguish between malignant and benign CLNs and might aid the decision of choosing between the watch-and-scan strategy and surgery in primary cases as well as between repeated surgery and short-term follow-up in oncological patients.

**Abstract:**

Background: Enlarged cervical lymph nodes (CLNs) can result from infection or malignancies, and a definitive diagnosis requires histological examination. Ultrasound (US) remains the first-line imaging modality for detection, and new US techniques may improve characterization. The aim of our study was to investigate whether the qualitative assessment of multiparametric US (mpUS) can improve diagnostic performance in the differentiation of benign and malignant CLNs. Methods: 107 CLNs in 105 patients were examined by preoperative mpUS consisting of B-mode US, color-coded duplex sonography (CCDS), shear wave elastography (SWE) and contrast-enhanced US (CEUS). US images were evaluated in consensus by two experienced US operators. Histopathological examination was used as reference standard. Results: SWE and CEUS combined showed the highest overall diagnostic performance (91% sensitivity, 77% specificity, 87% positive predictive value (PPV), 83% negative predictive value (NPV), 90% accuracy, χ^2^ (1) = 51.485, *p* < 0.001) compared to B-mode US and CCDS (87% sensitivity, 44% specificity, 73% PPV, 65% NPV, 73% accuracy χ^2^ (1) = 12.415, *p* < 0.001). In terms of individual techniques, SWE had higher specificity than B-mode and CCDS (71% sensitivity, 90% specificity, 92% PPV, 64% NPV, 78% accuracy, χ^2^ (1) = 36.115, *p* < 0.001), while qualitative CEUS showed the best diagnostic performance of all investigated US techniques (93% sensitivity, 85% specificity, 91% PPV, 87% NPV, 90% accuracy, χ^2^ (1) = 13.219, *p* < 0.001). Perfusion patterns, homogeneity, presence of necrosis, and malignancy differed significantly between malignant and benign CLNs (*p* < 0.001). Conclusions: SWE and CEUS can facilitate the differentiation of inconclusive CLNs when performed to supplement B-mode US and CCDS. MpUS may thus aid the decision between surgery and a watch-and-scan strategy in enlarged CLNs.

## 1. Introduction

Cervical lymph nodes (CLNs) can be enlarged due to infectious conditions or in the presence of metastasis from malignancies such as head and neck squamous cell carcinoma (HNSCC) or lymphatic diseases. Differentiation requires a histological examination [1]. Many HNSCCs first present with signs and symptoms of CLN metastasis [2], where CLN involvement is a prognostic factor and influences the choice of treatment [3,4]. Close monitoring of CLNs is recommended in HNSCC patients [5], with ultrasound (US) being the image modality of choice [6]. In B-mode US, benign CLNs have an oval shape, a well-defined border and a hilus sign. Deviations from any of these morphological features are considered indicators of potential malignant involvement [7]. Even though the combination of B-mode US and CCDS shows a higher diagnostic performance than computer tomography or magnetic resonance imaging of the neck, its sensitivity of 66–91% and specificity of 61–78% are not yet satisfying [6,8]. Recent studies have demonstrated the added value of utilizing preoperative quantitative multiparametric US (mpUS) in inconclusive CLNs [9,10,11]. MpUS has recently been developed to include multiple modalities such as shear wave elastography (SWE) and contrast-enhanced ultrasound (CEUS) in addition to regular B-mode US and color-coded duplex sonography (CCDS) [12]. SWE was found to allow differentiation of metastatic from benign CLNs based on metric evaluation of tissue stiffness, which resulted higher in malignancy [13]. Moreover, SWE was found to enable the distinction between malignant and benign CLNs even when none of the morphological B-mode US criteria were present [9]. Quantitative CEUS, which depicts CLN vessel structure and gives a real-time image of a tissue’s microvascularization, identified no significant differences between benign and malignant nodes [9,13]. This observation might seem counterintuitive given that most metastatic CLNs show a higher density of blood vessels [14] and, depending on their size, are also characterized by the presence of necrosis [15]. Thus, this study aimed to evaluate whether the qualitative assessment of mpUS images, which already were assessed quantitively [9], can improve diagnostic performance in the differentiation of benign and malignant CLNs.

## 2. Materials and Methods

### 2.1. Patient Selection

In this single-center study, we prospectively screened 105 eligible patients referred by the otorhinolaryngological department’s outpatient clinic between August 2019 and September 2021 using the following inclusion criteria: (a) age 18 or older, (b) enlarged CLN(s) for more than two weeks, (c) suspicious CLN(s) during oncological aftercare and (d) additional histological confirmation. Histological confirmation was obtained by analyzing samples of biopsy, lymph node dissection, neck dissection or US-guided core needle biopsy. US examinations were performed as part of presurgical workup using a standardized examination protocol. The study was approved by the institutional ethics committee of Charité-Universitätsmedizin Berlin (protocol code EA1/087/19, date of approval: 2 May 2019), and written informed consent was obtained from all participants according to the Declaration of Helsinki.

### 2.2. Imaging Protocol

All US examinations were performed by a trained otolaryngologist or an experienced radiologist using a single high-end US system with a 4–10 MHz multifrequency linear- array transducer and a center frequency of 7 Mhz (Acuson Sequoia, Siemens Healthineers, Erlangen, Germany). B-mode US of CLNs was performed to assess size, shape, echogenicity and homogeneity. CCDS was used to determine the vascularization of the nodes. SWE was performed using the 2D SWE approach.

**Figure 1:**
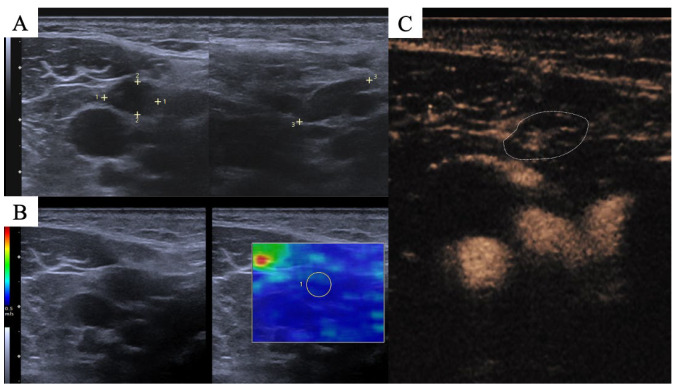
Representative example of ultrasound image evaluation. (**A**): B-mode US, CLNs were evaluated in two planes for their morphological appearance (size, shape, and borders; 1–3: diameter in each plane) (**B**): SWE, left side with B-mode US image and color bar (blue indicating soft and red indicating hard tissue), right side showing result of SWE assessment within the ROI (yellow circle marked with “1”). (**C**): Contrast inflow of a CLN at 15 s of a 90 s CEUS cine loop. The video was evaluated for perfusion pattern, distribution homogeneity and presence of necrosis.

Shear waves are generated by the ultrasound probe by using an acoustic radiation force pulse sequence. These waves spread vertically to the ultrasound beam, which causes tissue displacement. The velocity distribution of each shear wave at each pixel of the image is measured by the computer and relates to the shear modulus, which is known as the absolute measure of the elastic properties of tissue. The measured velocities are coregistered with the B-mode image and are depicted in a color-coded SWE-map [16]. Five consecutive images were obtained, and for the purpose of the quantitative assessment which was presented in a previous study [9], a circular region of interest (ROI) adapted to the CLN size was placed in dual-image mode for assessment of the whole CLN (B-mode image left, color-coded SWE map on the right, ROI for measurement visualized in both images). The color-coded SWE map represents CLN stiffness from soft to hard in blue, green, yellow and red as illustrated in Figure 1. To optimize visualization the standardized penetration depth was adapted to each patient while gain was kept steady in order not to influence SWE measurement.

The same high-end system was used for CEUS examinations. We used a state-of-the-art CEUS-specific protocol and a low mechanical index (<0.1) to avoid early microbubble destruction. A 90 s cine loop was recorded while a bolus of 1.2 mL of a second-generation ultrasound contrast agent (SonoVue^®^, Bracco Imaging, Milan, Italy) was injected intravenously followed by a 10 mL flush of 0.9% saline solution. The contrast agent consists of microbubbles filled with sulfur hexafluoride and is eliminated through the lungs within 10–15 min. It causes almost no adverse events (0.088%), with headaches or nausea representing the most recent mild adverse events (0.047%) [17].

### 2.3. Image Interpretation

All B-mode US images, CCDS and CEUS loops were retrospectively evaluated in consensus by an experienced radiologist and an experienced otolaryngologist (DEGUM (Deutsche Gesellschaft für Ultraschall in der Medizin, German Society of Ultrasound in Medicine and Biology) Level II in head and neck US). B-mode US features were homogeneity, CLN shape and borders [18]. Inhomogeneous and irregular-sized and -shaped CLNs were rated as malignant. CCDS findings were classified as central, peripheral or no hypervascularization; CLNs showing a hilus sign were rated as benign [19]. SWE was rated by interpreting the color-coded-SWE-map, where blue and light green were rated as benign and dark green, yellow and red were rated as malignant CLN. Please note that the ROI that was placed in every CLN was only used to assess the quantitative SWE-measures which are presented in [9]; these results are not part of this study. CEUS features included the contrast enhancement pattern and distribution homogeneity (centrifugal or centripetal pattern, general hypo- or hyperenhancement) and presence of avascular areas, which were rated as necrosis, based on the malignancy criteria described by Xiang et al. [20]. CLNs were rated by two readers and indicated malignancy when showing an irregular centripetal distribution pattern or necrosis [21,22]. MpUS (combination of all four parameters) was rated as malignant if one application showed a morphologic pattern of malignancy.

### 2.4. Statistical Analysis

Ultrasound findings and histopathological reports of each patient were analyzed retrospectively for diagnostic performance by testing sensitivity, specificity, positive predictive value (PPV) and negative predictive value (NPV) as well as accuracy using cross tables. A two-sided significance level of α = 0.05 was defined appropriate to indicate statistical significance. Relationships between categorical variables were tested using Pearson’s chi-square test. Group differences (malignant and benign histopathological result) in perfusion patterns or presence of necrosis were calculated using the Mann–Whitney U-test.

A univariate logistic regression of individual multiparametric ultrasound parameters was performed to assess the parameter’s association with predicting malignancy in CLN. All four parameters served as covariates in the multivariate logistic regression. All statistical analyses were performed using the SPSS software (IBM Corp. Released 2016. IBM SPSS Statistics for Windows, Version 27.0. Armonk, NY, USA: IBM Corp.).

## 3. Results

### 3.1. Study Population

Ultrasound findings obtained from 107 CLNs (39 benign, 68 malignant) among 105 patients were included (37 female, 68 males; mean age 59.93 years (standard deviation (SD) ± 16.59 years)). Surgical histological confirmation was available in 103 patients (either CLN removal or neck dissection; in two patients a target LN was taken from either side of the neck). Two patients could not undergo surgery due to medical reasons and therefore underwent US-guided core needle biopsy (14-gauge needle; Magnum™ Reusable Core Biopsy Instrument, minimum of three specimens). There were 68 malignant CLNs; the majority (30 CLNs, 44.12%) originated from HNSCC, followed by lymphoma (20 CLNs, 29.41%), malignant melanoma (9 CLNs, 13.24%), adenocarcinoma (5 CLNs, 7.35%) and other entities (4 CLNs, 5.88%). The results are compiled in Table 1.

### 3.2. Diagnostic Performance

#### 3.2.1. Diagnostic Performance of Single Modalities

The univariate logistic regression showed that all four parameters have a significant association to the prediction of malignant CLN (*p* < 0.001). For more detailed information see Table 2.

B-mode US showed a sensitivity of 87%, specificity of 46%, PPV of 74%, NPV of 67% and an accuracy of 72% (χ^2^ (1) = 14.236, *p* < 0.001), while CCDS had a sensitivity of 85%, specificity of 67%, PPV of 82%, NPV of 72% and accuracy of 79% (χ^2^ (1) = 29.974, *p* < 0.001) in differentiating malignant from benign CLNs.

SWE identified malignant CLNs with a sensitivity of 71%, specificity of 90%, PPV of 92%, NPV of 64% and accuracy of 78% (χ^2^ (1) = 36.115, *p* < 0.001).

CEUS allowed differentiation between benign and malignant CLNs with a sensitivity of 93%, specificity of 85%, PPV of 91%, NPV of 87% and an accuracy of 90% (χ^2^ (1) = 13.219, *p* < 0.001). The interpretation of perfusion patterns (centrifugal distribution indicating benign CLN and centripetal distribution pattern suggesting malignant CLN) showed a sensitivity of 65%, specificity of 72%, PPV of 80%, NPV of 54% with the accuracy being 67% (χ^2^ (1) = 64.605, *p* < 0.001). The sensitivity, specificity, PPV, NPV and accuracy of CEUS distribution homogeneity were 75%, 74%, 84%, 63% and 75% (χ^2^ (1) = 24.638, *p* < 0.001) and of necrosis 60%, 82%, 85%, 54% and 68% (χ^2^ (1) = 17.967, *p* < 0.001).

#### 3.2.2. Diagnostic Performance of Combined Parameters

The diagnostic performance of B-mode US and CCDS in combination resulted in 87% sensitivity, 44% specificity, 73% PPV, 65% NPV and an accuracy of 73% (χ^2^ (1) = 12.415, *p* < 0.001). 

The accuracy of SWE and CEUS combined was calculated at 90%. Their combination showed a sensitivity of 91%, specificity of 77%, PPV of 87% and NPV of 83% (χ^2^ (1) = 51.485, *p* < 0.001). 

Investigating the combination of B-mode US, CCDS and SWE resulted in a sensitivity of 96%, a specificity of 77% with 72% PPV and 82% NPV and an accuracy of 76% (χ^2^ (1) = 21.168, *p* < 0.001).

The combination of all four parameters (B-mode US, CCDS, SWE and CEUS combined) resulted in a sensitivity of 97%, specificity of 36%, PPV of 73%, NPV of 88% (χ^2^ (1) = 18.072, *p* < 0.001) and an accuracy of 83%. Details of the results are listed in Table 3.

CEUS was the only mpUS parameter showing a significant association with the prediction of CLN in the multivariate logistic regression (*p* < 0.001). For further details, see Table 4.

### 3.3. Perfusion Patterns, Homogeneity and Necrosis

Statistically significant group differences between benign and malignant CLNs were found for perfusion patterns (centrifugal distribution and centripetal distribution (U = 842.000, Z = −3.619, *p* < 0.001)), homogeneity (U = 671.500, Z = −4.940, *p* < 0.001), and presence of necrosis (U = 764.500, Z = −4.219, *p* < 0.001).

## 4. Discussion

**Figure 2:**
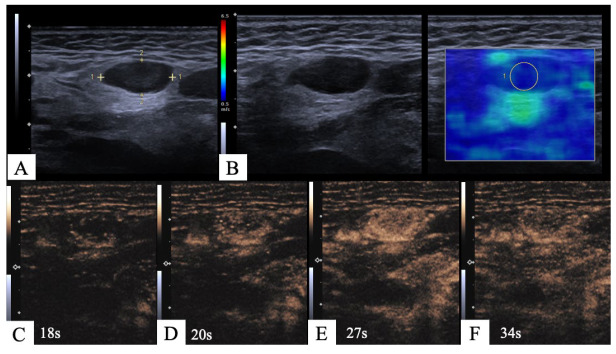
Metastatic CLN from HNSCC, only detected by CEUS and not by B-mode US or SWE. (**A**): B-mode US: oval shape, regular borders, and no criteria for malignancy. (**B**): SWE: B-mode US on the left; color-coded map of SWE on the right. Color bar on the left side with blue representing low and red representing high stiffness. Tissue in the ROI on the right side is blue and indicates soft tissue; therefore, there is no sign of malignancy. (**C**–**F**): CEUS 18, 20, 27 and 34 s after contrast agent administration. Contrast agent does not enter the node via a hilus but from the periphery (**C**,**D**). This is a centripetal perfusion pattern with subsequent short hyperenhancement in (**E**) and diffuse washout (**F**). These criteria suggest malignancy.

The results of our study show that complementing US examinations of CLNs by means of mpUS improves their diagnostic performance in qualitatively evaluating for the presence of metastatic involvement. The combination of SWE and CEUS as added modalities shows higher performance for all calculated parameters compared to the commonly used B-mode US and CCDS. In particular, CEUS-derived information on vascularization can help to differentiate between benign and malignant nodes. An example is presented in Figure 2.

CCDS and CEUS, which visualize vascular patterns, showed the highest sensitivity at 87% and 93%, respectively, and high PPV (82% and 91%) and accuracy (79% and 90%). This supports our hypothesis that altered vascularization resulting from changes in vessel density in malignant CLNs [14] can be visualized after administration of an ultrasound contrast agent, thus supporting the detection of malignant CLNs. Vascularization patterns differed significantly between malignant and benign CLNs. Benign CLNs were identified by showing a centrifugal enhancement pattern, which is characterized by a hilus sign, from where the contrast agent spread throughout the node, ultimately resulting in homogeneous enhancement. Smaller malignant CLNs showed a centripetal pattern in conjunction with hyperenhancement. This is in line with histopathological findings showing higher blood vessel density in metastatic CLNs within the scope of tumor neoangiogenesis than in reactively enlarged CLNs [14,23]. Tumor neoangiogenesis is characterized by an irregular vessel distribution throughout the node. In larger metastatic CLNs, we additionally observed non-enhancing areas, suggesting necrosis surrounded by an enhancing rim. During neoangiogenesis, vessels grow irregularly, which leads to an avascular center with rim enhancement [24]. This can be explained by the fact that tumor necrosis is not only associated with angiogenesis but also with hypoxia and inflammation [25]. One characteristic sign of inflammatory processes is rim enhancement, which is defined as a hypovascular area surrounded by a hypervascular border [26], for example in abscesses [27]. The differentiation of infectious from malignant processes as the source of this imaging phenomenon has to be made clinically (i.e., clinical presentation, blood tests) [24]. Rim enhancement in nodal metastasis is often seen in magnetic resonance imaging or computed tomography [28], but it is also visible in CEUS. A recent study has shown that rim enhancement in CEUS of axillary lymph nodes is associated with breast cancer metastasis [29]. These irregular vascular patterns highlight the value of additional qualitative assessment by the investigator, since quantitative time–intensity curve analyses performed by the perfusion software are not suitable in CLNs with larger areas of central necrosis (showing no dynamic perfusion in necrotic areas). This may explain why quantitative CEUS assessment showed no statistically significant difference between benign and malignant CLN in previous studies [9] and the discrepancy in our current study addressing qualitative assessment. In general, necrotic areas may be easier to detect through dynamic real-time CEUS examination without the need for quantitative assessment.

The transformation from benign to HNSCC-metastatic lymph nodes is not only associated with an increase in vascularization, but also leads to higher stiffness due to tumor cell infiltration [30,31]. This is corroborated by both our earlier quantitative SWE measurement, which showed higher shear wave velocity, and therefore higher stiffness in malignant CLNs compared to benign ones [9], and the qualitative evaluation of SWE in the current study. SWE achieved the highest diagnostic performance in terms of specificity (90% versus 46% for conventional B-mode US). Furthermore, SWE is the US technique with the highest PPV, followed by CEUS. During the US examination, the system displays SWE results in real time in a color-coded map of the distribution of tissue stiffness values in the ROI of the target area. Such maps allow easy and fast visual evaluation, making US-based SWE a feasible tool in the evaluation of inconclusive CLNs. One limitation of the examination is that there is no consensus whether to place one large ROI covering as much of the CLN as possible or if it is of advantage to use multiple smaller ROIs within the target lymph node. Further studies and a standardized training program are needed to establish a commonly used evaluation system which not only makes the comparison of study results easier but also ensures higher quality in US diagnostics [32].

Finally, it is important to highlight, that US examinations consist of the combined use of different modalities [33]. Our results do not only show the diagnostic value of SWE and CEUS as single but also as combined modalities. The addition of SWE to standard US (B-mode and CCDS) increased the accuracy from 73 to 76%, while adding CEUS to standard US increased the accuracy to 77%. Adding both new parameters to standard US led to an increase in diagnostic accuracy by 10%.

Overall, our results suggest a benefit of mpUS in the assessment of inconclusive CLNs, which can not only be used in initial diagnostic workup but also within follow-up proceedings after treatment. The high specificity of SWE suggests its usefulness in confirming benign CLNs, while the high sensitivity of CEUS makes it a useful parameter to further evaluate CLNs suggestive of malignancy. While the quantitative analysis of mpUS is time-consuming and, for CEUS, requires dedicated postprocessing software, a qualitative interpretation of findings is made by the operator during the US examination. SWE is a noninvasive and fast-to-perform (approximately 5 s per image) diagnostic tool requiring merely a specific ultrasound probe, while CEUS involves intravenous contrast agent administration, which necessitates prior preparation such as the placement of an intravenous line and an assistant who administers the agent and a saline flush. We therefore suggest the implementation of SWE as part of routine head-and-neck US and to complement the examination by CEUS in case of inconclusive SWE findings.

## 5. Conclusions

Measures of the diagnostic performance of the qualitative US assessment in CLNs showed that mpUS exceeds standard US techniques, driven especially by the high accuracy of mpUS. Moreover, mpUS is fast and easy to perform, underlining its potential for routine use on top of B-mode US and CCDS in the differentiation between benign and malignant CLNs, and might therefore aid the decision to choose between the watch-and-scan strategy and surgery in primary cases as well as between repeated surgery and short-term follow-up in oncological patients.

## Figures and Tables

**Table 1:** Study Population. Demographic data, CLN status, and primary cancer entities.

Patients	105
Female	37/105 (35.24%)
Male	68/105 (64.76%)
Mean Age	59.93 years (±16.59 years)
CLNs	107
Benign	39/107
Malignant	68/107
HNSCC	30/68 (44.12%)
Lymphoma	20/68 (29.41%)
Malignant melanoma	9/68 (13.24%)
Adenocarcinoma (breast, salivary gland, and lung)	5/68 (7.35%)
Other entities (prostate cancer, transitional cell carcinoma, atypical fibroxanthoma, and renal cell cancer)	4/68 (5.88%)

Continuous variables are given as mean (SD), categorical variables are given as absolute/total numbers (n/N) and percentages in brackets. Abbreviations: HNSCC = head and neck squamous cell carcinoma.

**Table 2:** Univariate logistic regression results of the individual multiparametric ultrasound parameters predicting malignant cervical lymph nodes. Abbreviations: US = ultrasound; CCDS = color-coded duplex sonography; SWE = shear wave elastography; CEUS = contrast-enhanced ultrasound; Coef(B) = standardized regression coefficient; S.E. = standard error; CI = confidence interval.

Variable	Coef(B)	S.E.	Odds Ratio	95% CI Lower–Upper	*p*-Value
B-mode US	1.1726	0.481	5.619	2.190–14.420	<0.001
CCDS	2.451	0.482	11.6	4.507–29.854	<0.001
SWE	3.045	0.591	21	6.593–66.891	<0.001
CEUS	4.238	0.643	69.3	19.67–244.149	<0.001

**Table 3:** Diagnostic performance of the qualitative assessment of different US techniques and parameters. Sensitivity, specificity, PPV, NPV and accuracy are given in percentages with corresponding *p*-values and χ^2^ (degrees of freedom stated in brackets).

	Sensitivity	Specificity	PPV	NPV	Accuracy	χ^2^	*p*-Value
Single Modalities							
B-mode US	87%	46%	74%	67%	72%	χ^2^ (1) = 14.236	<0.001
CCDS	85%	67%	82%	72%	79%	χ^2^ (1) = 29.974	<0.001
SWE	71%	90%	92%	64%	78%	χ^2^ (1) = 36.115	<0.001
CEUS	93%	85%	91%	87%	90%	χ^2^ (1) = 13.219	<0.001
CEUS Characteristics							
Perfusion pattern	65%	72%	80%	54%	67%	χ^2^ (1) = 64.605	<0.001
Homogeneity	75%	74%	84%	63%	75%	χ^2^ (1) = 24.638	<0.001
Necrosis	60%	82%	85%	54%	68%	χ^2^ (1) = 17.967	<0.001
Combined Modalities							
B-US + CCDS	87%	44%	73%	65%	73%	χ^2^ (1) = 12.415	<0.001
SWE + CEUS	91%	77%	87%	83%	90%	χ^2^ (1) = 51.485	<0.001
B-US + CCDS + SWE	96%	36%	72%	82%	76%	χ^2^ (1) = 18.072	<0.001
B-US + CCDS + CEUS	96%	38%	73%	83%	75%	χ^2^ (1) = 20.536	<0.001
mpUS	97%	36%	73%	88%	83%	χ^2^ (1) = 21.168	<0.001

Abbreviations: PPV = positive predictive value; NPV = negative predictive value; χ^2^ = Pearson’s chi-square test; US = ultrasound; CCDS = color-coded duplex sonography; SWE = shear wave elastography; CEUS = contrast-enhanced ultrasound; mpUS = multiparametric ultrasound.

**Table 4:** Results of multivariate logistic regression containing all four multiparametric ultrasound parameters predicting malignant cervical lymph nodes. Abbreviations: US = ultrasound; CCDS = color-coded duplex sonography; SWE = shear wave elastography; CEUS = contrast-enhanced ultrasound; Coef(B) = standardized regression coefficient; S.E. = standard error; CI = confidence interval.

Variable	Coef(B)	S.E.	Odds Ratio	95% CI Lower–Upper	*p*-Value
B-mode US	0.039	0.0859	1.039	0.193–5.592	>0.05
CCDS	0.331	0.938	1.393	0.221–8.762	>0.05
SWE	1.367	0.778	3.925	0.854–18.045	>0.05
CEUS	3.291	0.939	26.875	4.266–169.321	<0.001

## Data Availability

The datasets generated and analyzed during the current study are not publicly available but are available from the corresponding author on reasonable request.

## References

[1] Gaddey H.L., Riegel A.M. (2016). Unexplained Lymphadenopathy: Evaluation and Differential Diagnosis. Am. Fam. Physician.

[2] Johnson D.E., Burtness B., Leemans C.R., Lui V.W.Y., Bauman J.E., Grandis J.R. (2020). Head and neck squamous cell carcinoma. Nat. Rev. Dis. Primers.

[3] Audet N., Beasley N.J., MacMillan C., Jackson D.G., Gullane P.J., Kamel-Reid S. (2005). Lymphatic vessel density, nodal metastases, and prognosis in patients with head and neck cancer. Arch. Otolaryngol. Head. Neck Surg..

[4] Wunschel M., Neumeier M., Utpatel K., Reichert T.E., Ettl T., Spanier G. (2021). Staging more important than grading? Evaluation of malignancy grading, depth of invasion, and resection margins in oral squamous cell carcinoma. Clin. Oral. Investig..

[5] Paleri V., Urbano T.G., Mehanna H., Repanos C., Lancaster J., Roques T., Patel M., Sen M. (2016). Management of neck metastases in head and neck cancer: United Kingdom National Multidisciplinary Guidelines. J. Laryngol. Otol..

[6] Schneider U., Grass I., Laudien M., Quetz J., Graefe H., Wollenberg B., Meyer J.E. (2021). Comparison of Clinical Examination and Various Imaging Modalities in the Diagnosis of Head and Neck Cancer. Int. Arch. Otorhinolaryngol..

[7] Dudea S.M., Lenghel M., Botar-Jid C., Vasilescu D., Duma M. (2012). Ultrasonography of superficial lymph nodes: Benign vs. malignant. Med. Ultrason..

[8] Liao L.J., Lo W.C., Hsu W.L., Wang C.T., Lai M.S. (2012). Detection of cervical lymph node metastasis in head and neck cancer patients with clinically N0 neck-a meta-analysis comparing different imaging modalities. BMC Cancer.

[9] Lerchbaumer M.H., Wakonig K.M., Arens P., Dommerich S., Fischer T. (2022). Quantitative Multiparametric Ultrasound (mpUS) in the Assessment of Inconclusive Cervical Lymph Nodes. Cancers.

[10] Pehlivan M., Gurbuz M.K., Cingi C., Adapinar B., Degirmenci A.N., Acikalin F.M., Pinarbasli M.O., Colak E. (2019). Diagnostic role of ultrasound elastography on lymph node metastases in patients with head and neck cancer. Braz. J. Otorhinolaryngol..

[11] Dudau C., Hameed S., Gibson D., Muthu S., Sandison A., Eckersley R.J., Clarke P., Cosgrove D.O., Lim A.K. (2014). Can contrast-enhanced ultrasound distinguish malignant from reactive lymph nodes in patients with head and neck cancers?. Ultrasound Med. Biol..

[12] Kaneko M., Lenon M.S.L., Storino Ramacciotti L., Medina L.G., Sayegh A.S., La Riva Rincon A., Perez L.C., Ghoreifi A., Lizana M., Jadvar D.S. (2022). Multiparametric ultrasound of prostate: Role in prostate cancer diagnosis. Ther. Adv. Urol..

[13] Sidhu P.S. (2015). Multiparametric Ultrasound (MPUS) Imaging: Terminology Describing the Many Aspects of Ultrasonography. Ultraschall Med..

[14] Zenk J., Bozzato A., Steinhart H., Greess H., Iro H. (2005). Metastatic and inflammatory cervical lymph nodes as analyzed by contrast-enhanced color-coded Doppler ultrasonography: Quantitative dynamic perfusion patterns and histopathologic correlation. Ann. Otol. Rhinol. Laryngol..

[15] Yang G., Rao M., Ren J., Yang X., Wang J., Wu Y., Tao X. (2021). Determination of Cervical Lymph Nodes Metastasis and Extra Nodal Extension Status by Quantitative Assessment of Border Irregularity and Apparent Diffusion Coefficient in Patients with Tongue Squamous Cell Carcinoma. J. Comput. Assist. Tomogr..

[16] Taljanovic M.S., Gimber L.H., Becker G.W., Latt L.D., Klauser A.S., Melville D.M., Gao L., Witte R.S. (2017). Shear-Wave Elastography: Basic Physics and Musculoskeletal Applications. Radiographics.

[17] Li Q., Yang K., Ji Y., Liu H., Fei X., Zhang Y., Li J., Luo Y. (2023). Safety Analysis of Adverse Events of Ultrasound Contrast Agent Lumason/SonoVue in 49,100 Patients. Ultrasound Med. Biol..

[18] Rohan K., Ramesh A., Sureshkumar S., Vijayakumar C., Abdulbasith K.M., Krishnaraj B. (2020). Evaluation of B-Mode and Color Doppler Ultrasound in the Diagnosis of Malignant Cervical Lymphadenopathy. Cureus.

[19] Chammas M.C., Macedo T.A., Lo V.W., Gomes A.C., Juliano A., Cerri G.G. (2016). Predicting malignant neck lymphadenopathy using color duplex sonography based on multivariate analysis. J. Clin. Ultrasound.

[20] Xiang D., Hong Y., Zhang B., Huang P., Li G., Wang P., Li Z. (2014). Contrast-enhanced ultrasound (CEUS) facilitated US in detecting lateral neck lymph node metastasis of thyroid cancer patients: Diagnosis value and enhancement patterns of malignant lymph nodes. Eur. Radiol..

[21] Spiesecke P., Neumann K., Wakonig K., Lerchbaumer M.H. (2022). Contrast-enhanced ultrasound (CEUS) in characterization of inconclusive cervical lymph nodes: A meta-analysis and systematic review. Sci. Rep..

[22] Kunzel J., Brandenstein M., Zeman F., Symeou L., Platz Batista da Silva N., Jung E.M. (2022). Multiparametric Ultrasound of Cervical Lymph Node Metastases in Head and Neck Cancer for Planning Non-Surgical Therapy. Diagnostics.

[23] Liang X., Yang D., Hu J., Hao X., Gao J., Mao Z. (2008). Hypoxia inducible factor-alpha expression correlates with vascular endothelial growth factor-C expression and lymphangiogenesis/angiogenesis in oral squamous cell carcinoma. Anticancer. Res..

[24] Sharma A., Jaiswal A.A., Umredkar G., Barle R., Sharma N., Banerjee P.K., Garg A.K., Membally R. (2017). Lymph Node Central Necrosis on the Computed Tomography as the Predictor of the Extra Capsular Spread in Metastatic Head and Neck Squamous Cell Carcinoma. Indian. J. Otolaryngol. Head. Neck Surg..

[25] Bredholt G., Mannelqvist M., Stefansson I.M., Birkeland E., Bo T.H., Oyan A.M., Trovik J., Kalland K.H., Jonassen I., Salvesen H.B. (2015). Tumor necrosis is an important hallmark of aggressive endometrial cancer and associates with hypoxia, angiogenesis and inflammation responses. Oncotarget.

[26] Lee S., Kim S.H., Park H.K., Jang K.T., Hwang J.A., Kim S. (2018). Pancreatic Ductal Adenocarcinoma: Rim Enhancement at MR Imaging Predicts Prognosis after Curative Resection. Radiology.

[27] Yoon S.J., Yoon D.Y., Kim S.S., Rho Y.S., Chung E.J., Eom J.S., Lee J.S. (2013). CT differentiation of abscess and non-infected fluid in the postoperative neck. Acta Radiol..

[28] Ogura I., Oda T., Sue M., Sasaki Y., Hayama K. (2018). Comparison between squamous cell carcinoma and inflammatory diseases of the oral and maxillofacial region using gallium-67 scintigraphy with computed tomography and magnetic resonance imaging. Pol. J. Radiol..

[29] Guo Y., Song Q., Pan Q. (2021). Correlation analysis between rim enhancement features of contrast-enhanced ultrasound and lymph node metastasis in breast cancer. Am. J. Transl. Res..

[30] Tan S., Miao L.Y., Cui L.G., Sun P.F., Qian L.X. (2017). Value of Shear Wave Elastography Versus Contrast-Enhanced Sonography for Differentiating Benign and Malignant Superficial Lymphadenopathy Unexplained by Conventional Sonography. J. Ultrasound Med..

[31] Li L., Mori S., Sakamoto M., Takahashi S., Kodama T. (2013). Mouse model of lymph node metastasis via afferent lymphatic vessels for development of imaging modalities. PLoS ONE.

[32] Todsen T., Ewertsen C., Jenssen C., Evans R., Kuenzel J. (2022). Head and Neck Ultrasound—EFSUMB Training Recommendations for the Practice of Medical Ultrasound in Europe. Ultrasound Int. Open.

[33] Ahuja A.T., Ying M. (2005). Sonographic evaluation of cervical lymph nodes. AJR Am. J. Roentgenol..

